# Gastroparesis is associated with oxytocin deficiency, oesophageal dysmotility with hyperCCKemia, and autonomic neuropathy with hypergastrinemia

**DOI:** 10.1186/1471-230X-9-17

**Published:** 2009-02-25

**Authors:** Julia Borg, Olle Melander, Linda Johansson, Kerstin Uvnäs-Moberg, Jens F Rehfeld, Bodil Ohlsson

**Affiliations:** 1 Department of Clinical Sciences, Gastroenterology Division, Malmö University Hospital, Lund University, Lund, Sweden; 2 Department of Clinical Sciences, Hypertension and Cardiovascular Disease, Malmö University Hospital, Lund University, Lund, Sweden; 3 School of Life Sciences, University of Skövde, Skövde, Sweden; 4 Department of Animal Environment and Health, Swedish University of Agriculture Sciences, Skara, Sweden; 5 Department of Clinical Biochemistry, Rigshospitalet, University of Copenhagen, Copenhagen, Denmark

## Abstract

**Background:**

Gastrointestinal (GI) dysmotility and autonomic neuropathy are common problems among diabetics with largely unknown aetiology. Many peptides are involved in the autonomic nervous system regulating the GI tract. The aim of this study was to examine if concentrations of oxytocin, cholecystokinin (CCK), gastrin and vasopressin in plasma differ between diabetics with normal function and dysfunction in GI motility.

**Methods:**

Nineteen patients with symptoms from the GI tract who had been examined with gastric emptying scintigraphy, oesophageal manometry, and deep-breathing test were included. They further received a fat-rich meal, after which blood samples were collected and plasma frozen until analysed for hormonal concentrations.

**Results:**

There was an increase in postprandial oxytocin plasma concentration in the group with normal gastric emptying (p = 0.015) whereas subjects with delayed gastric emptying had no increased oxytocin secretion (p = 0.114). Both CCK and gastrin levels increased after the meal, with no differences between subjects with normal respective delayed gastric emptying. The concentration of vasopressin did not increase after the meal. In patients with oesophageal dysmotility the basal level of CCK tended to be higher (p = 0.051) and those with autonomic neuropathy had a higher area under the curve (AUC) of gastrin compared to normal subjects (p = 0.007).

**Conclusion:**

Reduced postprandial secretion of oxytocin was found in patients with delayed gastric emptying, CCK secretion was increased in patients with oesophageal dysmotility, and gastrin secretion was increased in patients with autonomic neuropathy. The findings suggest that disturbed peptide secretion may be part of the pathophysiology of digestive complications in diabetics.

## Background

Many diabetic patients exhibit gastrointestinal (GI) dysmotility and autonomic neuropathy. Gastroparesis is the most well-described of the GI abnormalities, but oesophageal dysmotility is also common [1-3]. The aetiology to the dysmotility is largely unknown. Vagal or autonomic neuropathy, myopathy and damage to interstitial cells of Cajal (ICCs) have been proposed [4-6], but otherwise pathological findings have been sparse [7]. As many peptide hormones are involved in the regulation of the GI physiology, disturbed secretion of these peptides might contribute to a dysfunction.

Oxytocin and its receptor have recently been reported to be expressed in the GI tract [8,9]. Oxytocin is released in response to a fatty meal [10], which stimulates gastric emptying [11,12], and administration of a receptor antagonist during the meal delays the gastric emptying [13]. Cholecystokinin (CCK) and gastrin are released from endocrine cells in the upper GI tract in response to food, and they influence relaxation of the lower oesophageal sphincter (LOS), gastric acid secretion, gastric emptying, growth of gastric mucosa, contraction of the gallbladder, pancreatic growth, enzyme secretion, and intestinal motility [14,15]. Earlier studies have reported increased CCK and gastrin concentrations in plasma from diabetics with autonomic neuropathy [16-20], whereas patients without neuropathy showed normal concentrations, irrespective of GI function [17,19-21]. Recently, receptors for vasopressin, a peptide hormone homologous to oxytocin, have also been found in the GI tract [22], and vasopressin has been shown to affect the electrical rhythm in the human stomach [23,24].

Although both oxytocin, CCK, gastrin, and vasopressin are involved in postprandial release and motility, the plasma concentrations of these hormones have not been thoroughly examined in relation to dysfunction in GI motility or autonomic neuropathy. The aim of the present study was therefore to examine whether the concentrations in plasma of these hormones from patients suffering from diabetes mellitus and GI symptoms are related to abnormalities in gastric emptying, oesophageal motility, and autonomic nerve function.

## Methods

This study was performed according to the Helsinki declaration and approved by the Ethics Committee of Lund University. All patients were given written informed consent before entering the study.

### Subjects

Consecutive patients at the Diabetes Clinic at Malmö University Hospital, who complained of symptoms from the digestive tract and were supposed to have gastroparesis, were invited to gastric emptying scintigraphy, oesophageal manometry, deep-breathing test reflecting the autonomic nerve function and continuous subcutaneous glucose concentration for 72 h. Patients with regular use of opiates and other drugs influencing GI motility or hormonal release, or a marked reduced renal function were excluded. In addition, patients with severe retinopathy were excluded as some examinations demanded good eyesight. Twenty patients (10 women) with stable metabolic control accepted to participate [25].

### Basal examination procedure

Gastric emptying scintigraphy was performed with the subjects in a semi-recumbent position as earlier described. A test meal was prepared by adding 30–50 Mbq of technetium-99-labelled tin colloid to an egg, which was whipped in a glass cup while being heated in a water bath until coagulated. The scintigraphy half time (T_50_) was identified from the point at which this tendency line crossed the 50% value. Measurements of radionuclide were corrected for decay according to Collins et al. [26]. T_50 _> 2 standard deviation (SD) of healthy controls (= 70 minutes) was considered abnormal [27].

Patients who fulfilled one or more pathological values in the oesophageal manometry of the following five criteria were considered to suffer from oesophageal dysmotility: 1/Absence of peristaltic contraction in the oesophagus, 2/Mean peristaltic contraction amplitude < 30 or > 200 mm Hg in the oesophagus, 3/Percentage of simultaneous, non-propulsive peristaltic waves in the oesophagus > 10, 4/Speed of the peristaltic wave < 3 or > 6 cm/sec in the distal oesophagus, 5/Resting pressure in the LOS < 10 or > 30 mm Hg. Normal peristaltic activity was defined as propulsive contraction waves with peak amplitudes between 30–200 mmHg and a speed between 3–6 cm/sec [28].

The expiratory/inspiratory (E/I) ratio was calculated from the mean value of the longest R-R interval during expiration and the shortest R-R interval during inspiration. This is an established test of vagal, parasympathetic nerve function [29]. All test results were expressed in age-related values and an age-related value below -1.64 SD was considered abnormal [30].

### Patient characteristics

Out of the 20 patients included, 19 were further included in the present study. All patients were insulin-treated; 17 with type 1 and 2 with type 2 diabetes. The 19 patients were investigated with gastric emptying scintigraphy, 17 with a deep-breathing test and 13 with oesophageal manometry. The reason that not all the patients performed all the examinations, depended on that the patients had to perform several different examinations, and thought it was too much to go through everything. Ten of 19 patients had delayed gastric emptying (221 [116.0–362.5] minutes compared to 35.0 [23.5–44.5] minutes in patients with normal emptying) and 7 out of 13 patients showed dysmotility of the oesophagus in the form of aperistalsis and/or simultaneous contractions. Nine of 17 showed abnormal expiration/inspiration (E/I) quote as a sign of autonomic neuropathy. There were no correlations between disturbances in GI or autonomic function (data not shown). The distribution of the GI symptoms, which did not differ between groups except for abdominal fullness which correlated to delayed emptying, are presented in an earlier study [25]. There was no difference in sex, age, duration of diabetes, HbA1c or glucose levels between the groups of normal and abnormal function of respective parameter [25].

When scrutinizing the medical records, one was found to suffer from pernicious anaemia, whereas all the others had normal values of haemoglobin and cobalamines.

### Experimental procedure

All subjects were fasted overnight. In the morning they were given a fat-rich meal containing 150 g cream and 150 g water. This generated 60 g fat and 561 kcal. This meal corresponds to the same content of fat as earlier used to evoke CCK and oxytocin secretion [10]. Blood samples were taken through an intravenous catheter 10 min and immediately before the meal, as well as 10, 20, 30, 45, 60, 90, 120, 150 and 180 min after the meal. All blood samples consisted of 8.0 ml whole blood drawn into iced heparinised tubes. The plasma was separated and frozen at -20°C immediate after the experiment.

### Plasma analyses

#### Oxytocin

Oxytocin levels were determined using Correlate-EIA™ Oxytocin Enzyme Immunoassay Kit (Assay designs, Inc. Ann Arbor, USA) according to the instructions from the manufacturer. Included on each plate were standards and controls as recommended. Plasma samples were diluted five times in assay buffer before analysis. The washing procedure was performed using an Anthos Fluido micro plate washer (Anthos Fluido, Salzburg, Austria) and the absorbance was read using a Multiscan (R) Ex micro plate photometer (Thermo Electron Corporation, Vantaa, Finland). The colour development of the samples was read at 405 nm with background correction at 620 nm. The Ascent software was used for creation of standard curves, curve fitting and calculation of concentrations (Ascent software version 2.6 for iEMS Reader MF and multiscan, Vantaa, Finland).

#### Cholecystokinin (CCK) and gastrin

The concentrations of CCK in plasma were measured using a highly accurate radioimmunoassay, as previously described [31]. The limit of detection for this assay is 0.1 pmol/l with intra-assay and interassay variations of less than 5% and 15%, respectively, at both 3.7 and 15 pmol/l concentrations. The concentration of gastrin in plasma was also assayed by radioimmunoassay using rabbit antibody 2604–8 [32] and ^125^I-iodinated human gastrin-17 as tracer [33]. The detection limit was 5 pg/ml and the interassay coefficient of variation was below 8% in the range 10–100 pg/ml.

#### Vasopressin

The concentration of vasopressin in plasma was analyzed by a radioimmunoassay described by Rooke & Baylis [34].

### Statistical analyses

The concentrations are given as median [interquartile ranges, IQR]. The basal value is the mean of the two samples taken before the meal. Peak value is the highest concentration measured in plasma after the meal. The area under the curve (AUC) was calculated for oxytocin, CCK and gastrin from all samples taken. The Mann-Whitney U test was used for comparisons between groups and the Wilcoxon signed test for comparisons between basal and peak levels within the group. The Spearman test was used for correlations between different parameters. P < 0.05 was considered statistical significant.

## Results

### Hormone concentrations in plasma in relation to gastric emptying

**Figure 1:**
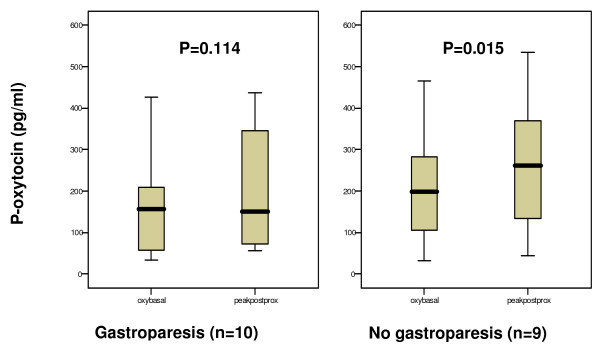
**The plasma concentration of oxytocin (oxy) at basal conditions and peakpostprandially, in subjects with and without gastroparesis**. The basal value is the mean of the two samples taken before the meal. Peak value is the highest level measured in plasma after the meal. Median [IQR]. Wilcoxon signed test. There was no difference in basal or peak values between the groups, Mann-Whitney U-test.

**Figure 2:**
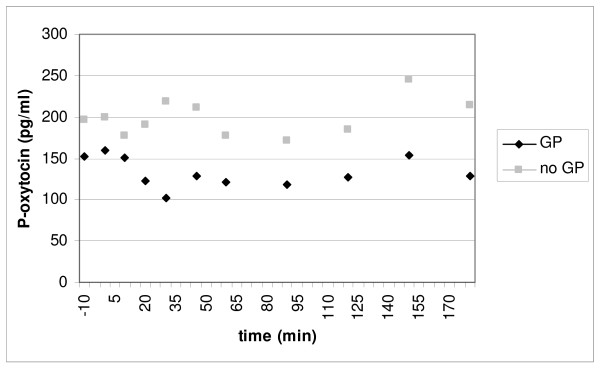
**The fluctuations of the mean oxytocin concentration in plasma postprandial, in subjects with and without gastroparesis**. GP = gastroparesis, n = 10, no gastroparesis, n = 9. There was no statistical significant difference between the groups. Mann Whitney U-test.

**Figure 3:**
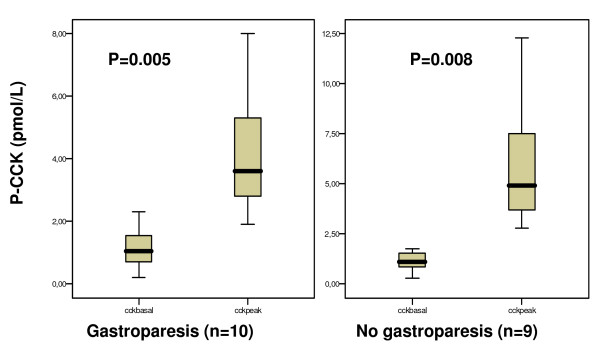
**The plasma concentration of cholecystokinin (CCK) at basal conditions and peakpostprandially, in subjects with and without gastroparesis**. The basal value is the mean of the two samples taken before the meal. Peak value is the highest level measured in plasma after the meal. Median [IQR]. Wilcoxon signed test. There was no difference in basal or peak values between the groups, Mann-Whitney U-test.

**Figure 4:**
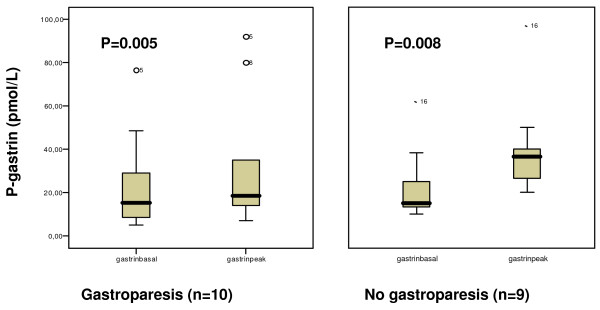
**The plasma concentration of gastrin at basal conditions and postprandially, in subjects with and without gastroparesis**. The basal value is the mean of the two samples taken before the meal. Peak value is the highest level measured in plasma after the meal. Median [IQR]. Wilcoxon signed test. There was no difference in basal or peak values between the groups, Mann-Whitney U-test.

There was a significant increase in postprandial oxytocin concentrations as compared to baseline in the group of patients with normal gastric emptying. In contrast, patients with delayed gastric emptying showed no increase of oxytocin secretion in response to the meal (Fig 1). However, the AUC did not differ between the two groups (16952.7 [8396.3–44792.2] and 37425.1 [14724.6–63779.9], respectively; p = 0.400), as did either basal or peak values (Fig 1). The oxytocin concentration in plasma in the two groups over time is shown in Fig 2. CCK and gastrin secretions were both increased compared to baseline in response to the meal. There were no differences in the secretion of these hormones between patients with normal versus delayed gastric emptying (Fig 3 and 4). The plasma concentration of vasopressin was not increased after the meal between patients with normal gastric emptying (0.7 [0.5–0.8] during basal conditions compared to 0.6 [0.5–1.0] pmol/l postprandial; p = 0.285), why this hormone was not studied in subjects with delayed gastric emptying. There were no significant correlations between the plasma concentrations of the different hormones (data not shown).

### Hormone concentrations in plasma in relation to oesophageal dysmotility

**Figure 5:**
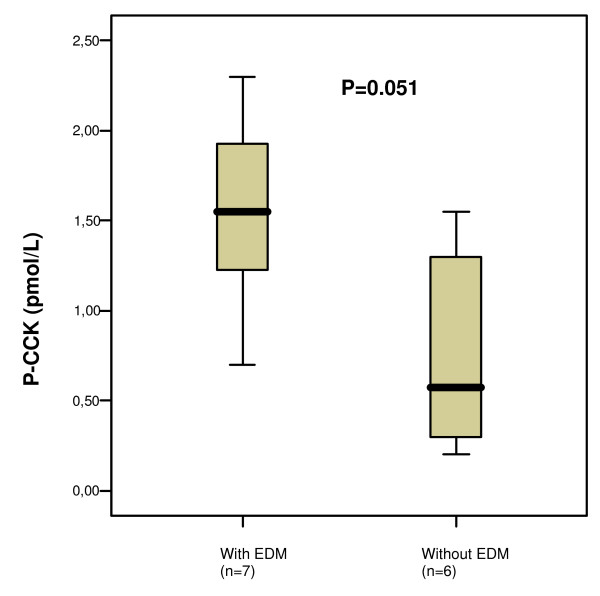
**The plasma concentration of cholecystokinin (CCK) at basal conditions, in subjects with and without oesophageal dysmotility (EDM)**. The basal value is the mean of the two samples taken before the meal. Median [IQR]. Mann Whitney U test.

The basal concentrations of CCK tended to be higher in the group of patients with aperistalsis and/or simultaneous contractions compared to those with normal oesophageal motility (Fig 5), whereas there was no difference in the peak concentrations measured after the meal (3.6 [2.8–5.3] and 3.4 [2.6–8.6] pmol/l, respectively; p = 0.604), nor in the AUC (377.8 [257.8–548.0] and 273.0 [230.6–419.2], respectively; p = 0.366).

The LOS pressure correlated negatively to the CCK concentrations (r_s _= -0.679, p = 0.022). None of the other hormones correlated with the oesophageal function (data not shown).

### Hormone concentrations in plasma in relation to autonomic neuropathy

**Figure 6:**
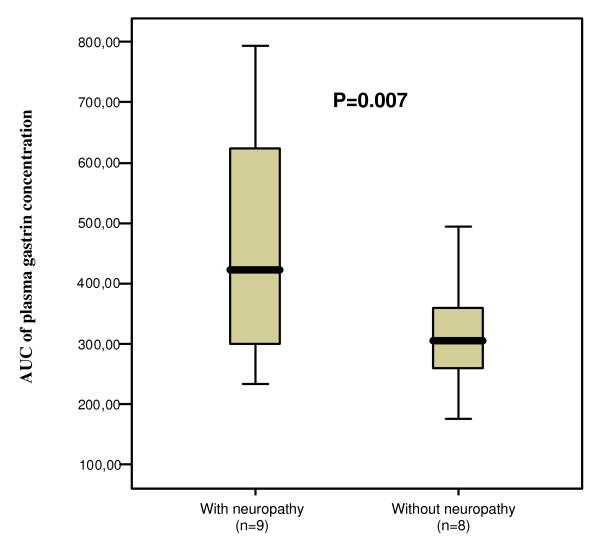
**Area under curve (AUC) of plasma gastrin concentration in subjects with and without autonomic neuropathy**. Median [IQR]. Mann Whitney U test.

Patients with autonomic neuropathy had significantly higher concentrations of gastrin in plasma than patients without neuropathy. There were differences both in the AUC (Fig 6) and the peak values (22.0 [15.5–57.0] and 13.5 [11.2–15.0] pmol/l, respectively; p = 0.046). There were no differences in oxytocin or CCK peak concentrations between the groups (181.5 [73.6–449.5] and 211.4 [139.7–346.8] pg/ml oxytocin, respectively; p = 0.888; and 4.4 [3.3–6.6] and 3.4 [2.8–5.6] pmol/l CCK, respectively; p = 0.423). Neither was there any difference in the AUC between those with or without autonomic neuropathy (29502.5 [9377.3–65353.4] and 30536.7 [10605.1–56321.3] for oxytocin, respectively; p = 0.743; and 423.0 [274.2–661.0] and 305.1 [258.4–369.0] for CCK, respectively; p = 0.195).

### Discussion

This is the first study to show that patients with diabetes mellitus and normal gastric emptying rate have an increased release of oxytocin postprandially, whereas patients with delayed gastric emptying rate have no such increased postprandial secretion. Instead, patients with delayed gastric emptying tended to have a decrease in oxytocin concentrations with less fluctuation over time. There was no difference in CCK or gastrin secretion related to gastric emptying, but patients with oesophageal dysmotility tended to have higher basal CCK concentrations, and in autonomic neuropathy gastrin concentrations were increased compared to normal subjects.

Oxytocin was long thought to be a hormone primarily involved in parturition and suckling. We now know that oxytocin has diverse effects throughout the human body, for example on the release of atrial natriuretic peptide and in endothelial and smooth muscle cells in blood vessels [35,36]. In normal subjects, oxytocin expression has been found in the gut [8,9], where it is secreted after a meal [10] and stimulates colonic activity [37]. It has further been demonstrated that systemic administration of oxytocin leads to enhanced gastric emptying [11,12] and that the oxytocin receptor antagonist atosiban delays gastric emptying significantly [13]. The prokinetic effect of oxytocin on the gut has been assumed to be similar to the one in uterine myometrium and mammary myoepitheal cells; i.e. intracellular release of Ca2+ which leads to muscle contraction via myosin light kinase activity [38].

Oxytocin concentrations were higher in this study than in earlier studies [10]. This depends on that ELIZA in general give higher values than radioimmunoasays (RIA). The two peaks in oxytocin secretion in diabetics without gastroparesis are similar to those seen in healthy subjects after a meal [10]. This could be explained by a rapid release of oxytocin from the gut and a later one from the hypophysis. The oxytocin release from the hypophysis is due to an effect of CCK on afferent vagal nerves and appears with some time delay after that the meal has induced CCK secretion [10,39]. This hypothesis is strengthened by the fact that this dual oxytocin release was not seen after intravenous CCK injection [10,40]. The reduced postprandial oxytocin secretion in subjects with delayed gastric emptying could be part of the pathogenesis in gastroparesis. It is still unknown whether this disturbance is primary or secondary to gastroparesis or depends on dysfunction in the CCK-oxytocin interaction, intrinsic gastric nerves, the vagal nerve, or in regulation of other hormones. Nevertheless, a substitution with oxytocin postprandially may regain the gastric dysfunction and should be tested.

Cholecystokinin is produced in endocrine I-cells in the upper part of the small intestine and has an important role in relaxation of the LOS and gastric emptying [14]. The oesophageal dysmotilities in patients in this study were aperistalsis and simultaneous contractions [25]. We do not know why they are associated with hyperCCKemia. It may be due to abnormal neural reflexes affecting the CCK release, or to concomitant small intestinal dysmotility delaying the clearing of nutrients from the small intestine with ensuing increase in CCK secretion [41]. In our earlier study we saw that patients with oesophageal dysmotility also had delayed blood glucose response after a meal [25], a finding in agreement with this discussion.

In a previous study Glasbrenner [19] found CCK to be elevated in plasma from patients with autonomic neuropathy. Another study showed significantly lower CCK concentrations in diabetics without autonomic neuropathy compared to healthy controls [42]. It is possible that the patients in Glasbrenner's study [19] with hyperCCKemia also suffered from oesophageal dysmotility, which could explain their results.

Gastrin is released in response to food intake and acts in the stomach by stimulation of acid secretion and mucosal growth [15]. This process is regulated by a negative feedback mechanism to prevent excessive acid secretion. In accordance with earlier studies we found that patients with autonomic neuropathy have significantly higher concentrations of gastrin in plasma [16-18,20]. The elevated gastrin concentrations cannot be explained by atrophic gastritis in this study since only one subject had this condition. However, the elevation could be a result of a disturbed feedback mechanism due to neuropathy in the stomach [6], vagal neuropathy [16,17], an increased quantity of gastrin cells in the stomach [43], reduced number of somatostatin cells [44], or hyperglycaemia [45].

Vasopressin is homologous to oxytocin. Recently it has been shown that the vasopressin receptor is expressed throughout the GI tract [22]. We measured the concentration of vasopressin after a meal in patients with normal gastric emptying and found no increased secretion compared to basal conditions. As vasopressin thus not seems to be an important peptide in relation to a meal, we decided not to measure vasopressin in the rest of the patients.

## Conclusion

We have shown that patients with diabetes mellitus and normal gastric emptying have a postprandial increase in oxytocin secretion, whereas oxytocin secretion is impaired in subjects with delayed gastric emptying. We could not find a difference in postprandial CCK or gastrin secretion in patients with and without delayed gastric emptying. However, in patients with oesophageal dysmotility the basal concentration of CCK tended to be higher, and in patients with autonomic neuropathy the gastrin concentrations were increased compared to normal subjects. In the light of our earlier research on oxytocin and its role for a normal GI function [11,12,33-37], the role of oxytocin treatment in gastroparesis has to be further evaluated.

## Abbreviations

AUC: area under the curve; CCK: Cholecystokinin; GI: gastrointestinal; ICCs: Interstitial cells of Cajal; LOS: lower oesophageal sphincter; SD: standard deviation; T_50_: scintigraphy half time.

## Competing interests

The authors declare that they have no competing interests.

## Authors' contributions

JB has substantial contributions to analysis and interpretion of data and mainly drafting the manuscript. OM has contributed to conception and design, acquisition of data, analysis and interpretion of data and has been involved in revising the manuscript critically. LJ, KUM and JFR have contributed to analysis and interpretion of data and have been involved in revising the manuscript critically. BO has contributed to conception and design, acquisition of data, analysis and interpretion of data and has been involved in drafting the manuscript. All authors have read and approved the final manuscript.

## Pre-publication history

The pre-publication history for this paper can be accessed here:

http://www.biomedcentral.com/1471-230X/9/17/prepub
